# Preparedness for infectious diseases during the Tokyo 2020 Olympic and Paralympic Games: advancing the health system beyond the games

**DOI:** 10.1016/j.lanwpc.2025.101488

**Published:** 2025-02-13

**Authors:** Chiaki Ikenoue, Munehisa Fukusumi, Satoshi Shimada, Tomoe Shimada, Motoi Suzuki, Yoshiyuki Sugishita, Tamano Matsui, Tomimasa Sunagawa, Tomoya Saito

**Affiliations:** a Center for Field Epidemic Intelligence, Research and Professional Development, National Institute of Infectious Diseases, Toyama 1-23-1, Shinjuku-ku, Tokyo 162-8640, Japan; b Center for Emergency Preparedness and Response, National Institute of Infectious Diseases, Toyama 1-23-1, Shinjuku-ku, Tokyo 162-8640, Japan; c Center for Surveillance, Immunization, and Epidemiologic Research, National Institute of Infectious Diseases, Toyama 1-23-1, Shinjuku-ku, Tokyo 162-8640, Japan; d Sumida City Public Health Center, Yokokawa 5-7-4, Sumida-ku, Tokyo 130-8628, Japan; e Infectious Disease Surveillance Center, National Institute of Infectious Diseases, Toyama 1-23-1, Shinjuku-ku, Tokyo 162-8640, Japan

**Keywords:** Mass gatherings, Enhanced surveillance, Public health preparedness, Infectious disease, Olympic and paralympic games, Health security, Risk assessment

## Abstract

Mass international gatherings pose significant health security challenges and demand robust preparedness for infectious diseases. Though demanding, this process can leverage heightened political and social attention to fortify core capacities. Despite Japan's advanced public health system for infectious diseases, there were still areas of vulnerabilities. Preparation for the Tokyo 2020 Olympic and Paralympic Games (Tokyo 2020) strategically enhanced the national system for infectious diseases through a three-step approach: (i) assessing risks, readiness, and gaps; (ii) addressing the identified gaps by strengthening or establishing systems; and (iii) performing operational exercises involving multiple stakeholders. COVID-19, which led to the postponement of Tokyo 2020, brought the strict public health measures taken during the event into focus. However, these primary conventional steps need to be further highlighted. Emphasizing their applicability beyond games time, this approach is a model for countries that host large-scale gatherings.

## Background

The periodic emergence of infectious diseases reminds us of the critical need to improve public health systems. However, transformations of systems are complex and seldom begin in advance, highlighting a gap between the acknowledged need for preparedness and the actual implementation of preemptive measures. Nevertheless, Japan proactively seized the opportunity to strengthen its national public health system for infectious diseases, in anticipation of the “Tokyo 2020 Olympic and Paralympic Games (Tokyo 2020)”.^1^

International mass gatherings pose unique public health challenges, such as unwelcoming weather conditions, natural disasters, and terrorism. Among them, infectious diseases have also been historically one of the main concerns.^2^, ^3^, ^4^, ^5^

The influx of many international travelers in a condensed timeframe increases the risk of importing infectious diseases that may be locally uncommon. Furthermore, the dense congregation increases the likelihood of outbreaks, and the dynamic movement of people can spread infections beyond the event’s immediate vicinity.^2^ Any severe case of disease associated with a mass gathering could attract significant social attention and potentially risk the host country’s reputation, especially if it impacts the event’s operations.^2^

In 2013, Tokyo was chosen to host the 2020 Summer Olympic and Paralympic Games (Table 1).^6^

**Table 1:** Timeline of the preparation for measures against infectious diseases during Tokyo 2020.

Year	Month	Events
2013	Sep	Tokyo was selected as the host city for the 2020 Olympic and Paralympic Games.^6^
	Oct	The government of Japan (GOJ) established “The Tokyo 2020 Olympic and Paralympic Games Promotion Office (Promotion Office) in Cabinet Secretariat.^7^^,^^8^
2014	Jan	Tokyo metropolitan government (TMG) established “Bureau of Tokyo 2020 Olympic and Paralympic Games Preparation (Tokyo 2020 Bureau).”^6^^,^^9^ “Tokyo Organising Committee of the Olympic and Paralympic Games (TOCOG)” was launched.^8^
2015	Feb	“Tokyo 2020 Games Foundation Plan” was formulated by TOCOG.^10^
	June	GOJ issued the act on special measures for the 2020 Tokyo Olympics and Paralympics.^7^^,^^8^
2017	Oct	National Institute of Infectious Diseases, Japan (NIID) published risk assessment on infectious diseases during the Tokyo 2020 Olympic and Paralympic Games period.^11^
2018	May	NIID started periodic release of a summary report of notified imported infectious disease cases in Japan.^12^
2019	Apr	Surveillance for undiagnosed serious infectious illness was implemented.^13^
	Apr	The first ministries meeting for measures against infectious disease towards Tokyo 2020 was held.^8^
	Aug	GOJ published the action plan to manage infectious diseases toward Tokyo 2020^14^
		The information-sharing system among the municipalities was started.^15^
	Dec	Measles and rubella vaccination campaign by GOJ started targeting Tokyo 2020 workforces and those potentially hosting travelers from abroad.^14^
2020	Jan	The World Health Organization (WHO) declared the COVID-19 outbreak as Public Health Emergency of International Concern (PHEIC).^16^ GOJ established the ad-hoc GOJ COVID-19 response headquarters (ad hoc GOJ HQ).^17^
	Mar	Decision to postpone Tokyo 2020.^18^
	Sep	Revised Tokyo 2020 games plan with COVID-19 countermeasures was released by TOCOG, the International Olympic Committee (IOC), and the International Paralympic Committee (IPC).^19^
	Dec	TOCOG and TMG announced the establishment of “The Tokyo 2020 health and hygiene support Tokyo Brunch (Tokyo HHB)” and “Infectious disease control centre, TOCOG” for ad-hoc taskforce sectors for COVID-19 during Tokyo 2020.^6^^,^^20^
2021	Feb	IOC/IPC and TOCOG released the first version of the “Playbook,” the Tokyo 2020 rule book for infection control among all participants.^21^
	Jul	GOJ declared a state of emergency for the Tokyo metropolitan area under the Act on Special Measures for Pandemic Influenza and New Infectious Diseases Preparedness and Response, considering its high occurrence of COVID-19 (from July 12th to August 22nd).^22^
	Jul–Sep	Tokyo 2020 held.^6^^,^^23^

Tokyo 2020: The Tokyo 2020 Olympic and Paralympic Games.

Drawing from the experiences of the past Olympic and Paralympic Games, such as those held in Rio de Janeiro in 2016 and London in 2012, Tokyo 2020 anticipated the convergence of several million international and domestic travelers during the event.^24^^,^^25^ As an international cosmopolitan city with around 14 million residents,^26^ the Tokyo metropolis has successfully hosted various mass gatherings, from cultural festivals and academic conferences to sporting events, including the 2002 FIFA Japan/Korea World Cup,^27^ the 2013 National Sports Festival,^28^ and the 2019 Rugby World Cup.^29^ However, these events were often held at a single or a few venues within the Tokyo metropolitan area and seldom extended beyond two consecutive weeks in the same city. Despite its abundant experience, Tokyo had not previously accommodated such a large influx of visitors from around the world across multiple venues simultaneously, spanning several months.

Regarding the exceptional scale and national significance of Tokyo 2020, the “Act on Special Measures for the 2020 Tokyo Olympic and Paralympic Games” was established in 2015 to contribute to smooth preparation and operation. This act established the “Tokyo 2020 Promotion Office (Promotion Office)” in the Cabinet Secretariat of the “Government of Japan (GOJ)”, which formulated basic policies, and special measures, including the use of national property.^30^ The groundwork for managing infectious diseases then began, involving public health agencies at the national and host city government levels.^23^

As the COVID-19 pandemic emerged during Tokyo 2020 preparations, an additional action plan became necessary. Though the unique COVID-19 response plan for Tokyo 2020 has been discussed in several studies,^31^, ^32^, ^33^ the primary preparedness for infectious diseases before the pandemic has rarely been highlighted.

Nonetheless, the insights gained from the Tokyo 2020 preparedness for infectious diseases prior to the pandemic offer practical lessons for enhancing the national health system. Given the rarity of hosting mass gatherings during a pandemic, these lessons are particularly beneficial for regions and countries hosting future large-scale events. Thus, this report reviews the methodological approach used in the preparations for infectious diseases for Tokyo 2020.

## Methods

### Outlining the main process of preparing for infectious diseases

The authors were the individuals responsible for preparing for infectious disease events during Tokyo 2020 within their respective public health agencies. By integrating their experiences, this study outlines the main process of preparing for infectious diseases from strategy development to the three-step approach through multiple stakeholder engagement.

### The original three-step approach

**Fig. 1:**
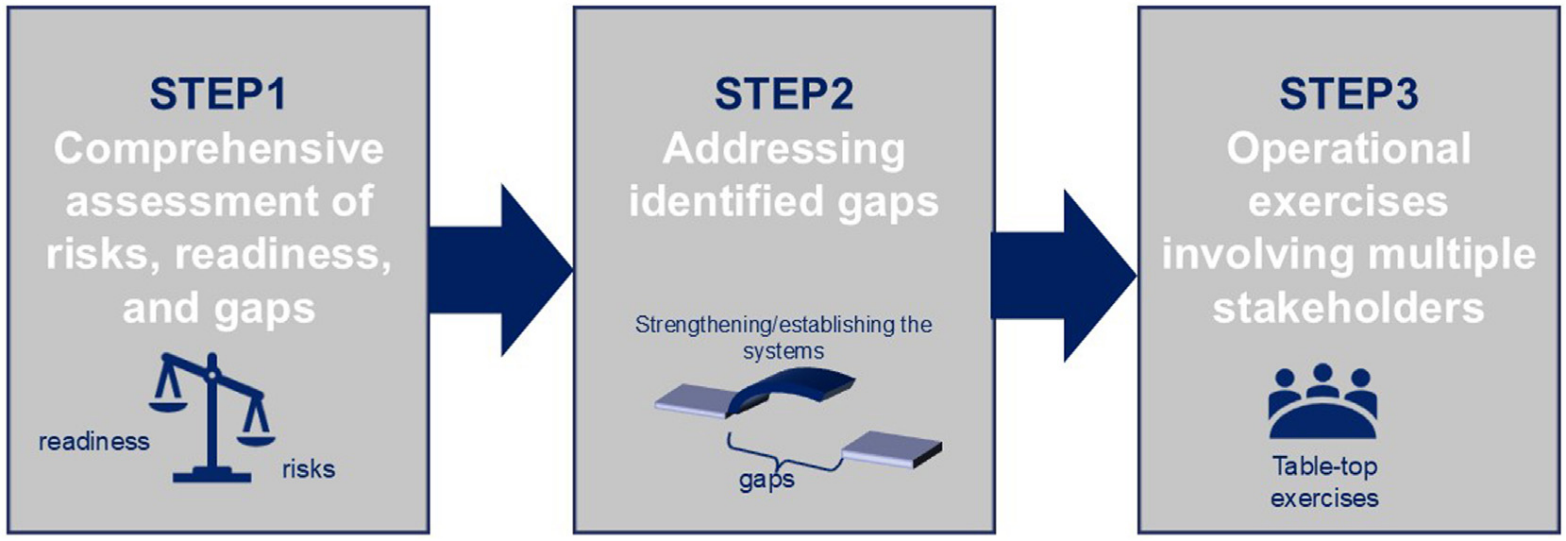
Three steps in public health planning for Tokyo 2020.

The original approach encompassed three steps: (i) assessing risks, readiness, and gaps; (ii) addressing the identified gaps by strengthening or establishing systems; and (iii) performing operational exercises involving multiple stakeholders (Fig. 1).

In the first step, infectious disease risks were categorized into four types: (a) increased risk of imported cases; (b) increased risk of transmission and a large outbreak; (c) risk of serious outcome; and (d) risk of increased burden on public health responses.^11^ According to each risk type, the gaps and readiness in the current system were examined. Subsequently, feasible strategies were devised to address them by the health authorities as the second step. Of the various procedures taken to bridge the gaps, principal initiatives that lasted after the games are described. The third step was the operational exercises involving multiple organizations to ensure seamless responses in the event of an infectious disease occurrence during the games period. Following the lessons from the past Olympic and Paralympic experiences, underscoring the critical importance of robust coordination among multiple organizations,^3^^,^^4^ the multi-stakeholder engagement through each of the steps was also described.

### The additional action plan for the emergence of COVID-19

Along with the emergence of the COVID-19 pandemic, how the process evolved to meet the increased demand to establish the COVID-19 response plan is described.

### Review of the policy documents

To verify the entire process, we thoroughly examined official reports, announcements, meeting minutes, and other relevant documents related to the preparation for infectious diseases. These documents were mostly issued by The Organising Committee for the Tokyo 2020 Olympic and Paralympic Games (TOCOG), GOJ, and the host city government, the Tokyo Metropolitan Government (TMG).

### Ethics approval

All data and information used in this study are publicly available; therefore, ethical approval was not required.

## Results

### Before the three-step approach: development of the fundamental strategy

In Japan, access to medical services is assured through a universal health insurance system, encompassing necessary laboratory tests to diagnose infectious diseases.^34^ With a laboratory diagnosis, it is mandatory for physicians to report notifiable diseases under the national surveillance program, which covers over a hundred infectious diseases. Based on these physicians’ notifications, local and national public health authorities can identify potential public health threats and subsequently take administrative control measures.^35^ This national surveillance system, regulated by law, stands as a pre-existing strength within Japan.

Recognizing the strength of existing systems for infectious disease, the governmental public health agencies and TOCOG collaborated to devise a cohesive policy for Tokyo 2020 as optimization of existing systems.^11^ The main governmental public health agencies involved included the “Bureau of Social Welfare and Public Health (BSWPH)” within TMG, the “National Institute of Infectious Diseases (NIID)”, and the “Ministry of Health, Labour and Welfare (MHLW)” of GOJ. To align with this policy, the public health agencies initiated a three-step approach.

#### Step 1: Comprehensive assessment of risks, readiness, and gaps

In 2017, NIID conducted an initial risk assessment of infectious diseases for Tokyo 2020. Based on this assessment, five diseases were evaluated as having high risks of multiple types: measles, rubella, invasive meningococcal disease (IMD), Middle East Respiratory Syndrome (MERS), and enterohemorrhagic *Escherichia coli* (EHEC).^11^^,^^36^ For example, measles presented a high risk of importation combined with considerable transmissibility, potentially leading to mass exposures during the game period. This could result in widespread, severe case occurrences and an urgent need for a bundle of public health responses, including close contact identification, health monitoring, vaccine procurement, and timely risk communications. Similarly, the other four diseases were associated with multiple risks. Consequently, these five diseases were regarded as priorities for which it was necessary to be well prepared in advance.

In addition to these five prioritized diseases, which can be diagnosed using existing schemes, any severe disease that cannot be diagnosed using existing practices was another recognized risk. Since the existing national surveillance system for infectious diseases was not flexible enough to capture “undiagnosed serious infectious illness,” some scheme needed to be developed to detect an event of an “undiagnosed serious infectious illness” that requires public health responses.

#### Step 2: Addressing the identified gaps through system enhancement or establishment

Recognizing the gaps in the current health system for infectious diseases, public health sectors formulated strategies responding to each gap, either by strengthening the existing system or establishing new ones (Table 2). Of various measures, five were the principal initiatives persisted afterwards.

**Table 2:** Comprehensive assessment of risks, readiness, and gaps.

Types of risks	Public health measures	Gaps in existing system	Approaches to address the identified gaps
**(i) Increased risk of imported cases** ***Diseases with potential risks*:** Tuberculosis, VPDs (Measles, Rubella, IMD, Influenza, Pertussis), Mosquito-borne diseases (Dengue, Chikungunya, Zika), MERS, Shigellosis, Hepatitis A/E, HIV/AIDS	Prevention of importation - Border measures - Vaccination before entering Japan	A quarantine system not sufficiently capable of coping with the rapid increase of inbound travelers.	Increasing the number of quarantine officers^a^^7^^,^^37^
		A quarantine system that cannot detect infectious disease carriers who are at risk of spreading infection after entering the country.	Developing pre-entry tuberculosis screening tests for those entering from tuberculosis-endemic countries.^38^
	Early detection and response - Raising awareness/knowledge among health care professionals and public health officials - Implementing a functional surveillance system - Conducting early diagnosis, ideally based on laboratory tests	Lack of knowledge and awareness among physicians and public health officials regarding infectious diseases with an elevated risk of importation.	Informing physicians and public health officials about the recent situation of infectious diseases outside Japan and imported infectious diseases through an easily accessible website.^a^^12^
		Lack of an applicable system to detect or diagnose infectious diseases brought in from outside the country by athletes or personnel related to the Tokyo 2020 Olympic and Paralympic Games.	Setting up the following systems for early detection of infectious diseases among athletes and personnel related to Tokyo 2020: - Syndromic surveillance at medical care facilities in the Tokyo 2020 games designated areas.^39^ - A medical hotline connecting to the medical task force in TOCOG.^39^ - Supportive collaboration by medical institutions, enabling the provision of diagnostic tests for personnel related to Tokyo 2020 suspected of having an infectious disease.^20^^,^^21^
**(ii) Increased risk of transmission and large outbreaks** ***Diseases with potential risks*:** VPDs (Measles, Rubella, IMD, Influenza, Pertussis), EHEC, Shigellosis, Hepatitis A/E, Acute gastroenteritis, Syphilis	Prevention - Raising awareness/knowledge among participants and related personnel of Tokyo 2020 - Promoting vaccination	Insufficient awareness among participants and related personnel of Tokyo 2020 about preventive measures against respiratory infectious diseases or food-borne diseases with increased risks at mass gatherings.	Advance health education for personnel related to Tokyo 2020 on general infection preventive measures, including hand hygiene and respiratory etiquette against infectious diseases with elevated risks at mass gatherings (i.e., respiratory diseases, food-borne diseases).^9^^,^^21^
		Vaccine coverage not achieving the targeted levels in some local areas, especially for VPDs with increased risks at mass gatherings (e.g., measles, rubella)	Strong recommendation for personnel related to Tokyo 2020 to undergo pre-event vaccination against VPDs with increased risks at mass gatherings (i.e., measles, rubella, IMD)^40^^,^^41^
	Early detection - Implementing a functional surveillance system - Conducting early diagnosis, ideally based on laboratory tests	No available system for timely information sharing among municipalities in the event of an infectious disease outbreak with a high risk of spreading over a jurisdiction.	Establishment of a timely information-sharing system among municipalities accessible via a secure intranet system for municipal health departments.^a^^15^
		No business continuity plan for events requiring laboratory tests that exceed the current capacity of medical institutions and local public health institutes.	Preparation for the swift shift from responding to all test requests to a sample test operation, in case of test requests exceeding the capacity of medical institutions and local public health institutes.^9^
		No applicable system for the timely detection of the accumulation of those suspected of having an infectious disease among athletes or personnel related to Tokyo 2020 and providing them laboratory tests for diagnosis.	Establish a system for timely testing and diagnosis of suspected infectious disease cases among athletes or personnel related to Tokyo 2020 through the implementation of the following systems: - Syndromic surveillance at medical stations in Tokyo 2020 security areas for early detection of cases - A hotline to the medical department of TOCOG for smooth consultation with the medical institute for laboratory tests.^20^^,^^21^
		No available system for timely information sharing among Tokyo 2020 stakeholders in the infectious disease outbreak with high risk of spreading among athletes or Tokyo 2020-related personnel.	Establishment of a timely information-sharing flow among Tokyo 2020 stakeholders in the event of an infectious disease occurrence in athletes or personnel related to Tokyo 2020 through the following process: - Sharing daily reports of infectious diseases among stakeholders^42^^,^^43^ - Use of smartphone applications for information sharing (i.e., Chat application, File-sharing system)^14^ - Daily cross-organizational meeting and urgent meetings on an as-needed basis.^42^^,^^43^
**(iii) Risk of severe outcomes** ***Diseases with potential risks*:** VPDs (Measles, IMD), EHEC, MERS	Early detection - Implementing a functional surveillance system - Conducting early diagnosis, ideally based on laboratory tests	No available system for the timely detection of an event of a severe case before laboratory diagnosis is confirmed. No existing scheme for public health agencies to implement epidemiological investigations and administrative measures for an event of severe disease, for which no laboratory diagnosis is available.	Establishment of an administrative scheme that allows public health responses, including epidemiological investigations, as well as laboratory tests by the government in the event of severe case, for which laboratory diagnosis was not made at medical facilities.^a^^13^
**(iv) Risk of increased burden on public health responses** ***Diseases with potential risks*:** VPDs (Measles, Rubella, IMD), EHEC, MERS	Public health responses - Epidemiological investigation - Conducting contact follow-up - laboratory testing by the government - Risk communication	Most public health centers are not sufficiently prepared for multilingual support in the event of an outbreak among athletes or personnel related to Tokyo 2020 who are subject to epidemiological investigations.	Use interpretation services and automatic translation equipment for multilingual support to facilitate smooth public health response.^a^^9^
		Concerns exist that people subject to public health responses, especially those from other countries, may not understand the administrative requests, such as epidemiological investigation or self-quarantine for those who were in contact with cases of infectious disease.	Inform athletes and personnel related to Tokyo 2020 in advance so that they can understand and cooperate with Japan's administrative response in the event of an infectious disease occurrence through the preparation of multilingual documents.^9^^,^^21^
		The operation for epidemiological investigation in the Tokyo 2020 security area, which should be promptly conducted by public health personnel on an as-needed basis, has not been clearly organized.	Organize operations among TOCOG and concerned administrative agencies to enable rapid epidemiological investigations in the Tokyo 2020 security area.^39^
		In the event of an outbreak or a rare severe disease among the athletes or personnel related to Tokyo 2020, special attention might be paid domestically and globally, potentially affecting the country’s reputation.	Organize procedures for conducting risk communication among stakeholders, including TOCOG, municipalities, national governments, and/or IOC/IPC, in preparation for the event of an outbreak or a rare severe disease among personnel related to Tokyo 2020.^20^
		Some municipalities that are not sufficiently prepared for simultaneous multiple events of infectious diseases or an event of a large-scale outbreak that require multiple epidemiological investigations and laboratory tests by the government.	Develop a scheme that allows the national government to provide prompt support when the burden on municipalities becomes overwhelming, including simultaneous responses needed for multiple events or a large-scale outbreak.^a^^42^

a Approaches that remained after the Tokyo 2020 games period. VPDs: Vaccine-preventable diseases. IMD: invasive meningococcal disease. MERS: Middle East respiratory syndrome. Hepatitis A/E: hepatitis A and hepatitis E. HIV/AIDS: human immunodeficiency virus infection/Acquired Immunodeficiency Syndrome. EHEC: Enterohemorrhagic *Escherichia coli*.

##### Initiative 1: Vaccination campaigns targeting high-risk populations

Of the five prioritized diseases, three were vaccine-preventable diseases (VPDs): measles, rubella, and IMD. For these VPDs, GOJ and TMG implemented vaccination campaigns. At previous mass gatherings, VPD measures were mostly vaccination recommendations by the health authorities, where implementation was up to the individuals. Compared with them, the vaccination campaigns by governmental agencies were the first effective VPD measures for mass gatherings.

###### Measles and rubella

Despite the achievement of measles elimination in 2015, Japan continued to face recurring outbreaks of measles cases, particularly around 2019, often triggered by imported cases.^44^ As for rubella, the male working-age population during the 2010s remained susceptible due to the past national vaccination policy, which targeted only females until 1980. Although the national measles and rubella (MR) vaccination campaign aiming at this rubella-susceptible male population was initiated by GOJ in 2019, vaccine uptake remained below expectations.^45^

The insufficient immunity against measles and rubella within the working-age population, especially among males, posed unignorable risks of outbreaks of these diseases in Tokyo 2020. Consequently, the GOJ planned an MR vaccination campaign focusing on individuals who anticipated having frequent contact with foreigners during Tokyo 2020. This included TOCOG workforce members, personnel in the tourism and transportation sectors, and persons hosting travelers from overseas.^37^^,^^40^

###### Invasive meningococcal diseases

As for IMD, which has the potential for rapid progression and high severity, awareness among healthcare workers (HCWs) in Japan was notably low due to the rarity of the disease in the country, with approximately 0.028 cases reported per 100,000 population per year.^46^ Even after the approval of the meningococcal vaccine in 2015, vaccine coverage remained low among HCWs. The TMG independently implemented a meningococcal vaccination campaign, considering the occasional reports of mass-gathering-related IMD events^47^ and the relatively higher risk for healthcare workers caring for patients from meningococcal disease-endemic areas. This campaign targeted HCWs who were candidates to work in Tokyo 2020 medical facilities. TOCOG supported this project.^41^

##### Initiative 2: A surveillance system for undiagnosed serious infectious illness (USII)

As highlighted in the risk assessment, detecting and responding to an undiagnosed serious infectious illness was recognized as a challenge for health authorities.

The reporting criteria of Japan's national infectious disease surveillance system usually mandate notification of cases when they exhibit signs/symptoms compatible with specific infectious diseases and/or are diagnosed through laboratory confirmation. Consequently, an event of severe illness that did not align with the existing reporting criteria may have been unnotified unless clinicians voluntarily provided the information. The absence of an administrative framework for clinicians to report such cases to government agencies hinders their timely detection.

To enable health agencies to respond promptly to severe yet unknown diseases, the MHLW modified the “syndromic surveillance system for suspected infectious diseases” into “Undiagnosed Serious Infectious Illness (USII) Surveillance” in 2019.^37^ This modified mechanism was designed for reporting severe illnesses that cannot be diagnosed through routine clinical practice at medical facilities.^13^ If any case was reported to this USII surveillance, public health authorities could perform further laboratory testing and initiate epidemiological investigations based on their risk assessment. The local authorities responsible for taking public health measures can request technical support from the NIID if necessary.

USII surveillance was primarily established to enhance the swift national response to USII, given that the illness may be a potential emerging disease. Furthermore, considering the heightened risks of uncommon illness events during international mass gatherings, the MHLW made announcements to promote the use of the USII surveillance during large mass gatherings in advance, as in the case of the 2019 Rugby World Cup^48^ and Tokyo 2020.^49^ Since the USII surveillance system was a unique scheme compared to the other surveillance systems for infectious diseases, in the way this required no specific case definition or laboratory test results upon notification, the MHLW and NIID collaborated to familiarize local health agencies and designated hospitals with this system by conducting training and workshops.

##### Initiative 3: Strengthening border control measures

Considering the potentially heightened risk of imported infectious diseases, the GOJ augmented quarantine officers at major airports as a border control measure in 2015. However, the gradual enhancement at borders was deemed insufficient to handle the surge in travelers in Tokyo 2020. In response, the GOJ significantly increased the size of the Customs, Immigration, and Quarantine (CIQ) staff by approximately 3000 members in preparation for Tokyo 2020.^7^^,^^37^

Despite the reinforced CIQ system at airports, preventing the entry of asymptomatic carriers of infectious diseases remained a challenge, with tuberculosis being a particular concern. The incidence rate of tuberculosis in Japan has consistently decreased since 2000, reaching 8·2 newly registered tuberculosis cases per 100,000 population as of 2022.^50^ To be certified as a country with a low incidence of tuberculosis (defined as less than 10 newly registered tuberculosis cases per 100,000 population), both national and local health authorities have invested considerable efforts to address the domestic spread of tuberculosis. The domestic incidence of tuberculosis decreased; however, the number of tuberculosis carriers entering Japan from tuberculosis-endemic countries who later developed symptoms increased.^7^ Regarding this situation, introduction of pre-arrival tuberculosis screening tests, specifically targeting travelers from tuberculosis-endemic countries had been discussed by the GOJ.^37^ Given the potential rise in tuberculosis carriers associated with the Tokyo 2020 period, GOJ promoted and organized this plan.^37^^,^^38^

##### Initiative 4: Raising awareness of imported infectious diseases

With the potentially increased risk of importing uncommon infectious diseases into Japan, concerns arose regarding the limited experience of public health officials and clinicians in managing such cases, potentially resulting in delayed diagnoses and a delayed public health response.

Recognizing that the extent of imported infectious diseases often reflects the prevalence of these diseases in their country of origin, trends in imported infectious diseases can serve as valuable tools for clinical diagnosis and epidemiological investigations. To enhance awareness of medical and health professionals regarding the trends in infectious diseases outside Japan that pose a risk of importation, in 2018, NIID initiated periodic surveillance reports on imported infectious diseases reported to the national surveillance system.^12^

##### Initiative 5: A timely information-sharing system among local governments in preparation for the spread of infectious diseases across jurisdictions

When an infectious disease spreads beyond a jurisdiction, epidemiological investigation and control measures can become complicated owing to the involvement of multiple stakeholders and the limitation of sharable information among them. Despite its complexity, there was a strong emphasis on timely information sharing to facilitate an early and broad public health response. However, no official, standardized means existed for a municipality responsible for the case patient to share confidential epidemiological information with another municipality before their epidemiological link was confirmed.

With the increased risk of widespread infectious events in Tokyo 2020 attributed to increased population movement across the country, the GOJ arranged an information-sharing system among local governments by using a shared folder function exclusively accessible to designated health officials in each local government. Originally, this system began for the 2019 Rugby World Cup^48^ and has been used on a weekly basis since then. For Tokyo 2020, this limited-access information-sharing system was again actively used, where NIID posted daily newly reported cases of the five prioritized diseases registered in the national surveillance system.^15^ The information provided by this system was restricted to non-personally identifiable data. MHLW and NIID encouraged local governments to adopt this system before Tokyo 2020 by updating the guidance specific to the games period. With this guidance, local health authorities across the country could receive daily updates on high-priority disease incidents during the games.^40^

#### Step 3: Operational exercises involving multiple stakeholders

According to the conventional contract between each Organising Committee for the Olympic Games (OCOG), the International Olympic Committee (IOC), and the International Paralympic Committee (IPC) for all Olympic and Paralympic Games, TOCOG had to establish a medical service system for the participating national delegates.^51^^,^^52^ This included the setup of polyclinics within the athletes’ village and medical stations within each venue and securing game-designated hospitals near each venue for further necessary medical care. The arrangement of this inclusive medical service for the game personnel required tremendous effort, and much was undecided within TOCOG in the earlier preparation phase. Although these uncertainties made operational planning for public health emergencies difficult, BSWPH of the TMG took the lead in conducting tabletop exercises in 2018 and 2019. The topics of the exercises were selected based on risk assessment and technical support provided by NIID.^53^

The participants included not only the main primary health agencies, BSWPH of TMG, NIID, and MHLW, but also TOCOG and another governmental sector specialized for Tokyo 2020: the “Bureau of Olympic and Paralympic Games Tokyo 2020 Preparation (Tokyo 2020 Bureau)” of TMG (Panel 1).^7^^,^^9^^,^^14^^,^^37^^,^^42^^,^^43^ Furthermore, the municipalities offering competition venues joined the exercises.^7^

Panel 1 **Stakeholders in Tokyo 2020 preparation.**

**Government of Japan (GOJ)**

**The Tokyo 2020 Olympic and Paralympic Games Promotion Office, Cabinet Secretariat (Promotion Office)∗**

A division, established by the Cabinet Secretariat of Japan, which was tasked with coordinating affairs within the purview of administrative departments to facilitate seamless preparation for the Tokyo 2020 Olympic and Paralympic Games. The establishment of the Promotion Office was stipulated in the “Act on Special Measures for the 2020 Tokyo Olympic and Paralympic Games” enacted in 2015.

**Ministry of Health, Labour and Welfare (MHLW)**

A national ministry, which advocates the comprehensive enhancement and advancement of social welfare, social security, and public health. “Health, Medical Care,” one of the MHLW’s core jurisdictions covers health, food safety, medical care, health insurance, pharmaceuticals and medical devices, and data-based health management initiatives. MHLW encompasses fields in “Children and Childrearing,” “Long-Term Care, Health and Welfare Services,” “Employment Security, Labour,” “Pension,” and others. For Tokyo 2020, the MHLW was responsible for coordinating the establishment, enforcement, and implementation of national-level public health responses, including nationwide vaccination campaigns, strengthening border measures, introducing surveillance systems, and risk communications.

**National Institute of Infectious Diseases (NIID)**

A national institute of MHLW, which serves the following functions: (1) research related to infectious diseases; (2) reference laboratory services for infectious diseases; (3) infectious disease surveillance; (4) national authorization and quality control of biological products and antibiotics; (5) international cooperation; (6) training provisions; and (7) outreach activities. Throughout the Tokyo 2020 games, NIID established the Emergency Operations Center (EOC) to enhance surveillance and support investigations to infectious diseases associated with Tokyo 2020. The EOC played a pivotal role in coordinating communication on the enhanced surveillance with key stakeholders, including the Organising Committee for the Olympic and Paralympic Games (TOCOG), Tokyo Metropolitan Government, local municipalities, and other national government ministries, through daily meetings and sharing daily surveillance reports.

**Tokyo Metropolitan Government (TMG)**

**•Bureau of Tokyo 2020 Olympic and Paralympic Games Preparation (Tokyo 2020 Bureau)∗**

A bureau in TMG, which was established in 2014 through the reorganization of the Bureau of Sports Promotion, which was in charge of promoting Tokyo’s sports administration. The Tokyo 2020 Bureau was set up to ensure preparation for the Tokyo 2020 games.

**•Bureau of Social Welfare and Public Health (BSWPH)**

A bureau of TMG, which is responsible for fields such as health promotion and health policy, medical policy, infectious disease control, food and drug safety, sanitation of the living environment, animal welfare, supporting medical care system building, social welfare for children and families, elderly people, disabled people, and development of welfare infrastructure. For Tokyo 2020, the BSWPH was responsible for managing responses to infectious diseases in the Tokyo metropolitan areas and overseeing coordination among key entities such as the Tokyo Metropolitan Institute of Public Health, public health centers, MHLW, NIID, and agencies associated with Tokyo 2020, including TOCOG, Tokyo 2020 Bureau, and the Promotion Office.

**•Public health centers (PHCs)**

Municipal administrative agencies, which are responsible for conducting infectious disease surveillance, epidemiological investigations, implementing preventive measures, and providing guidance on health promotion and hygiene in response to infectious diseases.

**•The Tokyo 2020 Games Health and Hygiene Support Tokyo Branch (Tokyo HHB)∗**

A taskforce, which was established to assume an administrative role in responding to COVID-19 cases and other infectious diseases among the Tokyo 2020-related personnel. The Tokyo HHB conducted epidemiological investigations and health monitoring, instructing athletes and game-related personnel staying in athletes’ villages on preventive measures for infectious disease events. Tokyo HHB, composed of officials from the BSWPH, aimed to work in cooperation with the Infectious Disease Control Centre (IDCC) of TOCOG.

**Other municipalities**

**•Division of Health, Local Health Department**

A municipal department, which was responsible for responding to infectious diseases in the municipality, in coordination with the municipal institute of public health, public health centers, other municipalities, MHLW, NIID, and TOCOG.

**Organizations/Facilities designated with specific functions for Tokyo 2020∗**

**•Host towns∗**

Designated locations, which offered pre-game training camp sites and accommodations to Tokyo 2020 participating athlete delegates, typically organized by the delegates' countries and participating sports/disciplines. The sites and accommodations provided were predominantly existing facilities in the area, with local municipalities supporting the hosting of delegates. Cultural exchange events between the local residents in the host town and visiting delegates may have occurred during their stay.

**•Games designated hospitals∗**

Local hospitals, which were designated to provide medical care to Tokyo 2020-accredited personnel, including athletes, referees, and Tokyo 2020-related VIPs. TOCOG established contracts with these hospitals before the Tokyo 2020 games to ensure prompt and appropriate medical services for game participants.

**The Organising Committee for the Olympic and Paralympic Games Tokyo 2020 (TOCOG)**

The event organizer of Tokyo 2020, established in 2014.

**•Main operation centre (MOC)∗**

A headquarters within TOCOG, which was responsible for organizing the overall event operations and coordination within and outside TOCOG. This involved interactions with various relevant organizations, including the International Olympic Committee (IOC), the International Paralympic Committee (IPC), the National Olympic Committees of participating countries, international sports federations, sponsor companies, and national and local governments in Tokyo and other municipalities.

**•Infectious diseases control centre (IDCC)∗**

A newly established division within TOCOG, which functioned as a task force to address COVID-19 events among Tokyo 2020 games-related personnel. This division was responsible for organizing infection control measures for COVID-19 cases, monitoring signals of infectious disease occurrence within Tokyo 2020-designated sites through Tokyo 2020 specific surveillance systems, and coordinating communication with health-related organizations outside TOCOG. This included Tokyo HHB, BSWPH, health divisions of other municipalities, NIID, and MHLW.

**•The Polyclinic in the Olympic and Paralympic village∗**

A medical facility, which was set up in the Olympic and Paralympic villages located in Tokyo, where most athletes and team officials were accommodated during the game period. This facility primarily provided medical care to athletes and team officials of national delegations, offering services such as internal medicine, dental care, and physical therapy. Its operation was supported by numerous healthcare providers from various medical institutions in Japan through a rotational schedule. Rapid diagnostic tests for SARS-CoV-2 were available, particularly for febrile patients in polyclinics. Laboratory diagnostic tests for other infectious diseases could be conducted in commercial laboratories under contract with TOCOG.

**•Medical stations∗**

Designated sites, which provided first-aid at each venue and satellite village. Most medical stations, especially at venues, were not equipped with diagnostic tests for infectious diseases.

∗Newly established organization/facility for Tokyo 2020.

These exercises helped stakeholders realize the necessary preparations, including the establishment of a surveillance system within TOCOG to monitor any event occurrence, communication channels between TOCOG and public health agencies, and the operation of field investigations in security areas, such as the athlete village and at each venue.

### Throughout the three-step approach: engaging multiple stakeholders

Engagement of multiple stakeholders required acceleration as the game period approached. Close communication with TOCOG, a newly launched organization with limited personnel experienced in public health, was especially essential for operational planning. To support TOCOG, the health authorities each assigned a few technical experts a few years before the games, who served as liaisons across organizations. Another key advantage of multi-organizational collaboration was the Field Epidemiology Training Program (FETP), which was established within NIID in 1999^54^ to train field epidemiology specialists. The involvement of FETP alumni in infectious disease response efforts across TMG, NIID, and TOCOG facilitated smoother cross-organizational communications through their pre-existing network.

Despite effective communication among technical personnel across organizations, challenges persisted in fostering understanding within each organization. In particular, the headquarters of TOCOG, TMG, and GOJ, as well as the Tokyo 2020-dedicated departments in each municipality, which were newly launched divisions, prioritized their original tasks over infectious disease prevention and control.

### An additional measure with the emergence of COVID-19

At the end of 2019, when most of the games' preparation process was accelerating, COVID-19 emerged and rapidly spread globally, leading GOJ, TMG, TOCOG, and IOC/IPC to decide to postpone Tokyo 2020 in March 2020.^18^ This decision made GOJ, TMG, and TOCOG urgently develop a COVID-19 response plan for the rescheduled games a year later. Since this was the first time in history that the Olympic and Paralympic Games were held during a pandemic, careful coordination continued at the top level of involved organizations.^55^

While preparing for the postponed games, the COVID-19 situation worsened domestically and globally, during which strict social measures such as the “stay home policy” were sporadically implemented. In response to these situations, stern COVID-19 countermeasures for Tokyo 2020 were developed with the following basic policies: (i) a substantial reduction in the number of individuals involved in the games, including no spectators for most competitions; (ii) stringent behavioral restrictions among game-related personnel; (iii) early detection of illness through daily health monitoring; and (iv) frequent screening tests for SARS-CoV-2 of game-related personnel. These policies were detailed in a rulebook named the “Playbook,” to which all game-related personnel were required to adhere.^21^

Before the emergence of COVID-19, challenges persisted in the feasibility of communicating infection prevention measures to game-related personnel and the uncertainty of self-reporting when individuals associated with the games became sick. However, this Playbook swiftly resolved these issues by mandating compliance from all participants.

The combination of “the daily health reporting system for all participants” and “the syndromic surveillance system at game-related medical facilities” was the main health-related signal monitoring system, established within TOCOG. Subsequently, a rapid response plan for COVID-19 was formulated. In this plan, task force teams were established within both TOCOG and TMG, the “Infectious Diseases Control Centre (IDCC)” and the “Tokyo 2020 Games Health and Hygiene Support Tokyo Branch (Tokyo HHB),” respectively,^6^ to begin initial health responses for COVID-19 events involving game-related personnel. The establishment of these task force teams aimed to avoid additional burdens on local medical and public health systems that had already been strained since the onset of the pandemic.

The IDCC was positioned within the main operation center, the headquarters of TOCOG, facilitating cross-sectoral responses within TOCOG.^33^^,^^39^ This was a significant improvement over concerns during preparations before the pandemic.

Health personnel from the TMG, NIID, and TOCOG maintained their collaboration by sharing the workspace among the task force teams, exchanging daily reports among each organization, and participating in daily online meetings.

During the games, sporadic COVID-19 cases were continuously detected under the influence of domestic and global surges of COVID-19. This necessitated timely epidemiological investigations and tireless efforts by the IDCC and Tokyo HHB. In response, the NIID and MHLW immediately initiated support for epidemiological investigations alongside the IDCC and Tokyo HHB.^39^^,^^42^

Before the postponement of Tokyo 2020, health authorities of TMG and GOJ tried to develop outbreak response frameworks at their respective levels.^43^ The feasibility of rapid multi-stakeholder engagement remained questionable until the end, but the COVID-19 pandemic prompted all stakeholders to realize its urgent need.

## Discussion

To prepare for infectious diseases during the Tokyo 2020 period, TMG and GOJ held on to the fundamental strategy to maximize the existing systems and proceeded with a three-step approach. Even in the Japanese regulated health system for infectious diseases, various vulnerabilities were identified. GOJ took advantage of the momentum of this event when decision makers moved the system refinement forward.

For the various health risks associated with Tokyo 2020, including heat-related illness, chemical pollution, and natural disasters, existing systems and the event-specific countermeasures were considered sufficient to address potential incidents.^7^^,^^56^ In contrast, infectious diseases were exceptional in that they required updates to the existing systems.

When reviewing the procedure for the three-step approach, Step 2 was achieved well without extra burden on local municipalities. However, given the experience of Tokyo 2020 during the pandemic, the extra burden of the host city public health responses should have been assessed higher by the local and national health authorities than they had expected during Step 1. Furthermore, operational adjustment, which should have been developed after Step 3, was paused, owing to the urgent need to develop COVID-19 measures. While there are some points to reflect on, this three-step approach firmly promoted preparedness for Tokyo 2020.

The strict measures against COVID-19 for Tokyo 2020 attracted social attention,^28^^,^^43^^,^^57^ which were carried on to the Beijing 2022 Olympics held the following year. Yet, now that Tokyo 2020 and Beijing 2022 have concluded, and the Public Health Emergency of International Concern (PHEIC) phase of COVID-19 has ended, the lasting efforts were the refined public health systems in line with the initial policy for Tokyo 2020 preparedness.

The WHO demonstrated a framework for strengthening health systems through promoting six components: (i) service delivery; (ii) health workforce; (iii) information; (iv) medical products, vaccines and technologies; (v) financing; and (vi) leadership and governance.^58^

Reflecting on the preparations for infectious diseases for Tokyo 2020 using this WHO Building Blocks framework, we can confirm that each initiative contributed to advancing the health system for infectious diseases. The two vaccine campaigns provided valuable lessons for service delivery and raised awareness of the targeted diseases among healthcare workers and event personnel. The USII surveillance system became a crucial information system for detecting the initial COVID-19 cases in Japan and served as a reporting mechanism until a dedicated system was established.^59^ With the gradual lifting of border measures following the end of the COVID-19 PHEIC phase, the enhanced CIQ, implemented by the strong leadership of the GOJ, launched the genome surveillance of infectious diseases at the airports in 2023.^60^ As inbound visitors returned to the country, surveillance reports on imported infectious diseases provided travelers and healthcare providers with reliable information regarding sporadic spread of these diseases. The information-sharing system for municipalities is prepared for future outbreaks that could extend beyond the jurisdiction. Though further discussion of the scope of sharable information may be needed, this system will enhance the use of health information. Cross-organizational communications between TMG and NIID, which had undergone daily communications during Tokyo 2020,^42^^,^^43^ were preserved in response to other emerging events. The cross-organizational communications and implementations of these common initiatives for Tokyo 2020 motivated public health officials nationwide to recognize the importance of infectious disease preparedness for mass gatherings, contributing to the enhancement of human resources. Although the “medical products” and “financing” set aside for this event were temporary, the other five components were significantly strengthened through preparedness for Tokyo 2020.

Thus, we believe that advancing the three-step approach through effective communication among multiple organizations can pave the way to prepare for infectious diseases for mass gatherings and further improve the pre-existing system.

In 2025, Japan will host the 25th Summer Deaflympics in Tokyo and the World Exposition in Osaka, marking more large-scale international gatherings. Preparation for these events has begun with the relevant organizations. Through the same three-step approach, the systems introduced for Tokyo 2020 were evaluated and refined as needed.

After years of struggling with the challenges posed by the COVID-19 pandemic, countries are in a phase of reviewing and reconstructing their respective public health systems.^61^^,^^62^ The lessons derived from the Tokyo 2020 preparation process not only represent the resilience of Japan but also offer applicable insights for upcoming hosts of mass gatherings. These insights extend beyond the responsibilities of hosting events and encompass broader considerations of public health preparedness.

## Contributors

T Saito, MF, and CI were responsible for the design and drafting. T Saito, MF, and CI accessed to raw data, and verified the data, the released reports, and shared records provided by Tokyo 2020 stakeholders. YS, TM, and SS provided input and expertise in the relevant sections. MS, T Sunagawa, and T Shimada contributed to the overall manuscript structure and concept. T Saito had final responsibility for the decision to submit for publication.

During the preparation of this work the authors used ChatGPT-4 to improve language and readability. After using this tool/service, the authors reviewed and edited the content as needed and take full responsibility for the content of the publication.

## Declaration of interests

T Saito was a member of the Scientific Experts Round Table for COVID-19 Countermeasures established by TOCOG and an advisor to the Coordination Meeting for COVID-19 Countermeasures at the Olympic and Paralympic Games Tokyo 2020. CI, MF, and T Shimada contributed as technical experts for TOCOG during preparation and game time. SS contributed to the Tokyo 2020 preparation as a member of the MHLW until 2019, was also a member of TOCOG since 2020, and served as the chief of the public health response team of the IDCC. This report does not represent the opinions of the organizations to which the authors belong. All the authors declare no conflicts of interest.
